# DNA replication licensing and cell cycle kinetics of normal and neoplastic breast

**DOI:** 10.1038/sj.bjc.6602829

**Published:** 2005-11-08

**Authors:** A Shetty, M Loddo, T Fanshawe, A T Prevost, R Sainsbury, G H Williams, K Stoeber

**Affiliations:** 1 Department of Pathology, University College London, Rockefeller Building, University Street, London WC1E 6JJ, UK; 2 Wolfson Institute for Biomedical Research, University College London, The Cruciform Building, Gower Street, London WC1E 6BT, UK; 3 Centre for Applied Medical Statistics, Department of Public Health and Primary Care, University of Cambridge, Forvie Site, Robinson Way, Cambridge CB2 2SR, UK; 4 Department of Surgery, Royal Free and University College Medical School, University College London, Charles Bell House, 67-73 Riding House Street, London W1W 7EJ, UK

**Keywords:** Ki67, Mcm2, MCM, geminin, DNA replication licensing, breast cancer

## Abstract

Mcm2–7 (MCM) proteins are part of the origin licensing machinery that regulates initiation of DNA replication. Geminin is a licensing repressor and prevents reinitiation of DNA replication during S–G2–M phase by blocking reloading of Mcm2–7 at replication origins. Here, we have analysed these replication licensing factors (RLFs) to determine whether the pathway becomes deregulated during mammary carcinogenesis, and have assessed their potential value as prognostic markers. Protein expression profiles were generated for Ki67, Mcm2, geminin, HER-2, ER and PR in a series of reduction mammoplasty (*n*=18) and breast cancer specimens (*n*=120), and compared to clinicopathological parameters. A large proportion of epithelial cells of the terminal duct lobular unit reside in a primed ‘replication licensed’ but not proliferating state. This state is characterised by Mcm2 expression and absence of Ki67 and the S/G2/M marker geminin. In breast cancers, increasing tumour grade is associated with increased Ki67, Mcm2 and geminin expression. The Mcm2/Ki67 ratio decreases through the grades, indicating a shift from a predominantly licensed state to an actively proliferating state. This shift is associated with an increase in the geminin/Ki67 ratio, signifying a shortening of G1 phase in breast cancer cells. Ki67, Mcm2 and the Mcm2/Ki67 ratio are statistically significantly associated with the Nottingham Prognostic Index (NPI), but geminin and the geminin/Ki67 ratio are not. Ki67, Mcm2 and Mcm2/Ki67 are highly correlated with one another, with Mcm2 being the single most important predictor of NPI score (*P*<0.001). However, only 12% of variation in NPI is explained by Mcm2, as the labelling index for this marker is approaching 100% for many of the high-grade tumours. The origin licensing phenotypes of normal breast and breast cancers therefore relate to their cellular differentiation status, and high-level MCM expression in more poorly differentiated tumours severely constrains their use as prognostic markers in breast cancer.

With one million new cases in the world each year, breast cancer is the most common malignancy in women and comprises 18% of all female cancers. In the UK, breast cancer accounts for more than 14 000 deaths each year and the incidence is increasing with a prevalence of nearly 2% (McPherson *et al*, 2000). In spite of being one of the most aggressive cancers in women worldwide, there are encouraging signs that improvement in the mortality rate may be possible through earlier diagnosis and improved therapeutic interventions (Mori *et al*, 2002). Breast cancer is a complex disease due to its morphological and biological heterogeneity, its tendency to acquire chemo-resistance and the existence of several molecular mechanisms underlying its pathogenesis (Mori *et al*, 2002). Half of the women who receive loco-regional treatment for breast cancer will never relapse, whereas the other half will eventually die from metastatic disease (Bundred, 2001). It is therefore imperative to distinguish clearly between these two groups of patients for optimal clinical management. Unfortunately, prognostic markers for breast cancer are at present limited. The most widely used prognostic indicator is the Nottingham Prognostic Index (NPI), which combines the traditional and most important independent predictors of outcome including tumour size, histological grade and lymph node stage (Rampaul *et al*, 2001).

The initiation of DNA synthesis is a final and critical step in growth control and is therefore of importance in carcinogenesis. Initiation of eukaryotic DNA replication is dependent on the assembly onto chromatin of prereplicative complexes (pre-RCs) containing the origin recognition complex (ORC), Cdc6, Cdt1 and Mcm2–7 proteins (Bell and Dutta, 2002), thereby rendering origins licensed for one round of DNA replication during S phase (Mendez and Stillman, 2000; Blow and Hodgson, 2002; Dimitrova *et al*, 2002). Pre-RCs are activated by cyclin dependent kinases (CDK) and the Cdc7/ASK kinase, leading to recruitment of elongation factors, Cdc45, DNA polymerases and RPA to origins (Masai and Arai, 2002; Nishitani and Lygerou, 2002). The recruitment of these factors results in unwinding of the DNA helix and initiation of DNA synthesis (Lei and Tye, 2001). Replication initiation is tightly coupled to removal of the license, thus preventing re-licensing after origin firing. This step is critical, as origins must fire once and only once in each cell cycle to ensure genomic stability. To avoid re-licensing, mammalian cells have adopted a number of mechanisms. These include the inactivation of licensing factors during S–G2–M phase, a process controlled by CDK activity, regulated proteolysis and changes in gene expression. A further important mechanism is the repression of origin licensing by geminin (Nishitani and Lygerou, 2002). This repressor acts by competitively binding to Cdt1, thereby blocking Mcm2–7 re-loading onto chromatin (McGarry and Kirschner, 1998; Wohlschlegel *et al*, 2000; Quinn *et al*, 2001; Tada *et al*, 2001). Using membrane elution, we have demonstrated for human cells that geminin expression is restricted to the S, G2 and M phases of the cell cycle, in keeping with its function of preventing re-replication (Eward *et al*, 2004; Wharton *et al*, 2004).

The constituents of the pre-RC can be regarded as relay stations coupling growth regulatory pathways with DNA replication, thereby serving as novel biomarkers of growth (Williams and Stoeber, 1999). We have shown that repression of origin licensing is a ubiquitous route by which the proliferative capacity of metazoan cells is lowered during exit from the mitotic cell cycle. Withdrawal from cycle into quiescent (G0), differentiated or senescent states is coupled to downregulation of Cdc6 and MCM proteins. We have also demonstrated in a range of different tumour types that deregulation of Mcm2–7 is an early event in tumourigenesis, and have exploited these novel biomarkers of growth in primary diagnosis, surveillance and prognosis (Williams *et al*, 1998, 2004; Stoeber *et al*, 1999, 2001, 2002; Meng *et al*, 2001; Wharton *et al*, 2004). These studies have shown that the superior sensitivity of the MCM proteins over the standard proliferation marker Ki67 resides in the fact that Mcm2–7 identify not only cycling cells, but also noncycling cells with proliferative potential (Stoeber *et al*, 2001). Interestingly, this particular replication phenotype (Ki67 negative but MCM positive) is exhibited by premenopausal breast (Stoeber *et al*, 2001) and primary oocytes (Eward *et al*, 2004), resting tissues that retain proliferative capacity and that can respond rapidly to growth stimuli.

Here we have investigated regulation of Mcm2 and geminin in normal premenopausal breast to more precisely define the cell cycle kinetics of this unusual tissue type, which undergoes rapid periodic expansion. We have also analysed deregulation of the replication licensing pathway during mammary tumourigenesis. Expression of Mcm2 and geminin has been compared with receptor status (HER-2, ER, PR) and the standard proliferation marker Ki67 to investigate coupling between DNA replication licensing and differentiation. Expression profiles of these new molecular markers have been compared to clinicopathological parameters to assess their prognostic value.

## MATERIALS AND METHODS

### Clinical specimens

Patients who had surgery for invasive breast cancer were identified by a search of the histopathology records at University College London, UK. Archival formalin-fixed, paraffin-embedded breast tissue was retrieved from the archives of the Department of Pathology for 120 cases of invasive breast cancer, which included all three histological grades (1–3) calculated according to the Nottingham modification of the Bloom and Richardson method (Elston and Ellis, 2002). Histological reports and slides were available for all cases. These included 93 invasive ductal carcinomas, 20 lobular, four mucinous and three of mixed type. The parameters studied were histological grade, tumour size, tumour type, lymph node status, lymphovascular invasion (LVI), age and NPI. We also studied randomly selected cases of normal breast tissue from 18 women, 17 of whom had undergone reduction mammoplasty and one a prophylactic mastectomy for BRCA1. Local research ethics committee approval was obtained from the joint UCL/UCLH Committees on the Ethics of Human Research.

### Antibodies

Mouse anti-human monoclonal Mcm2 antibody (clone 46) was obtained from BD Transduction Laboratories™ (Lexington, KY, USA). Mouse anti-human monoclonal Ki67 (clone Mib-1), mouse monoclonal oestrogen receptor-α (clone 1D5) and mouse monoclonal progesterone receptor (clone PgR 636) antibodies were obtained from DAKO (Glostrup, Denmark). Affinity-purified rabbit polyclonal antibody G95 was generated against human geminin as described (Wharton *et al*, 2004).

### Immunohistochemistry

Sections (3 *μ*m) of formalin-fixed, paraffin-embedded tissues were cut onto DAKO TechMate™ S2024 charged slides, baked in a 60°C oven overnight to maximise section adhesion, dewaxed in xylene, and rehydrated through a series of alcohol to water. For antigen retrieval, tissues were pressure-cooked for 2 min in 0.1 M citrate buffer at pH 6.0. For ER, PR, Ki67, Mcm2 and geminin detection, automatic immunostaining was performed on a DAKO TechMate™ 500 as described (Dogan *et al*, 2000). Endogenous peroxidase activity was quenched by incubating the sections with peroxidase blocking solution (DAKO, S2023) for 10 min. Sections were then washed twice using Tris-buffered saline containing 0.1% Tween-20 (SIGMA, St Louis, USA) for this and subsequent washes. Slides were incubated with primary antibodies for 1 h at room temperature using the following concentrations: ER (1/100), PR (1/50), Ki67 (1/50), Mcm2 (1/1000), geminin (G95) (1/1000). After washing, antigen-bound primary antibodies were detected with a labelled streptavidin biotin detection system (DAKO ChemMate™ K5001). Slides were incubated with biotinylated secondary antibody (Ab2) for 30 min. After washing sections were incubated with streptavidin horseradish peroxidase for 30 min. After washing, the immunostain was developed applying 3,3-diaminobenzidine tetrahydrochloride (DAB) for 7 min. HER-2 immunostaining was performed using the DAKO HercepTest™ (DAKO K5205), according to the manufacturer's instructions. Slides were counter-stained with Mayer's haematoxylin, differentiated in 1% acid alcohol, dehydrated and cleared in xylene. Coverslips were applied with Leica CV Mount (Leica, Nussloch, Germany). Primary antibodies were omitted in negative controls and, in addition, appropriate tissue sections were used as positive and negative controls.

### Protein expression profile analysis

Slides were examined and 3–5 fields were captured with an Olympus BX51 microscope/CCD camera setup using ANAlysis software (SIS, Münster, Germany). Captured images were printed for quantitative analysis, and the labelling index (LI) for each marker was calculated as the percentage of cells stained positive. Cells were identified as positive if there was any nuclear staining present and any stromal or inflammatory cells on the field were excluded. A median of 400 cells was counted per slide. For evaluation of HER-2 protein overexpression, membrane staining was assessed following the criteria recommended by DAKO. Slides were examined by three independent investigators (GHW, ML and AS). GHW is a consultant histopathologist and re-examined all cases analysed by ML and AS. This assessment of interobserver variability showed high concordance.

### Statistical analysis

Different statistical techniques were used to compare the expression of markers to grade, lymph node metastasis, tumour size, NPI, and LVI. The Jonkheere–Terpstra test was used to examine whether there was any association between marker expression and grade. This is a nonparametric test, which takes into account the ordering of the categories of grade. A logistic regression model was used to analyse the relationship between marker expression and lymph node metastasis. Spearman's rank correlation coefficient was used to examine the association between marker expression and tumour size. Linear regression was used to model the relationship between marker expression and NPI. A Mann–Whitney test was used to examine the association between marker expression and LVI.

## RESULTS

### DNA replication licensing and cell cycle kinetics of normal resting breast tissue

**Figure 1:**
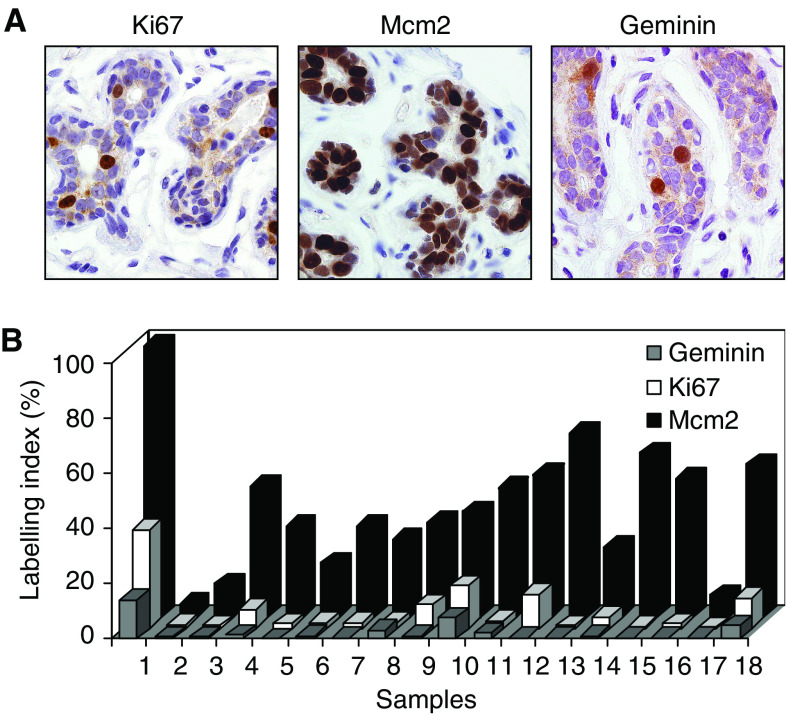
(**A**) Photomicrographs of paraffin-embedded tissue sections of normal premenopausal breast immunohistochemically stained with antibodies against Ki67, Mcm2 and geminin (original magnification, × 400). A high proportion of epithelial cells of the terminal duct lobular unit express Mcm2, a subpopulation Ki67 and only a minority geminin. The higher labelling index for Mcm2 compared to Ki67 reflects an additional replication licensed but nonproliferating growth fraction, identified by Mcm2 but not the standard proliferation marker Ki67. (**B**) Ki67, Mcm2 and geminin labelling indices for individual cases of normal premenopausal breast. Case 1 showing high levels of Ki67, Mcm2 and geminin expression is a patient who had a prophylactic mastectomy for BRCA1. All other cases of reduction mammoplasty show higher expression of Mcm2 compared to Ki67 and geminin.

We first studied expression of Mcm2, geminin, Ki67, and the ER and PR hormone receptors in a series of normal breast specimens (*n*=18). As reported by ourselves and others (Stoeber *et al*, 2001; Gonzalez *et al*, 2003), high-level Mcm2 expression was observed in epithelial cells of the terminal duct lobular unit (TDLU) in normal resting premenopausal breast (Table 1, Figure 1A and B). Whereas the level of Mcm2 expression was high, Ki67 was expressed at low levels (median Mcm2: 35.32% (range 4.9–98.43) compared to Ki67: 2.14% (range 0.22–35.32)). Geminin is present in only a minority of cells in premenopausal breast (median: 0.76% (range 0–13.87)). These data are in keeping with a high proportion of mammary epithelial cells residing in a licensed MCM expressing but nonproliferating state (Stoeber *et al*, 2001).

### DNA replication licensing and cell cycle kinetics of breast cancer

**Figure 2:**
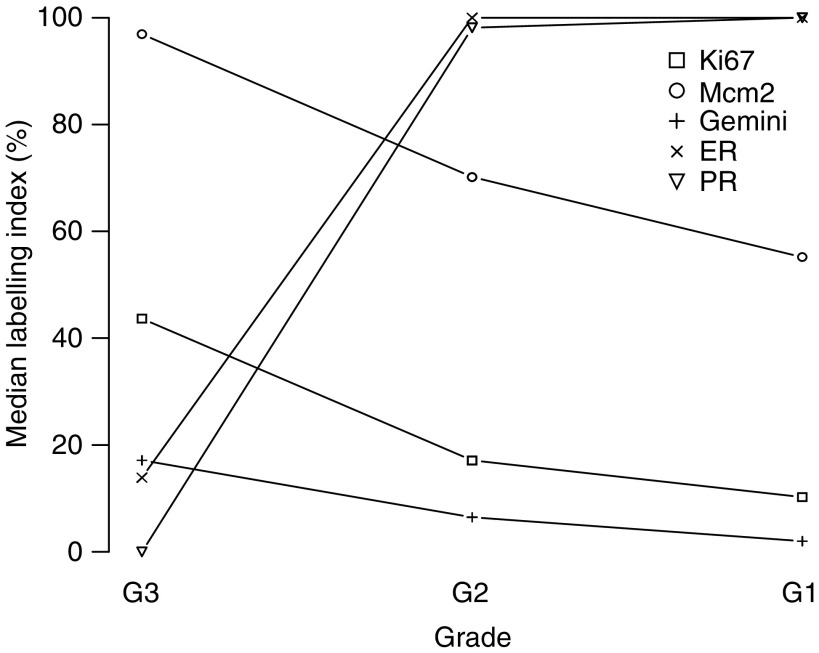
Ki67, Mcm2, geminin, ER and PR labelling indices (median percentage of cells stained positive) depicted across breast cancer grades 1–3. Increasing differentiation (from grade 3 to grade 1) is associated with decreasing Ki67, Mcm2 and geminin expression and increasing ER and PR labelling indices.

**Figure 3:**
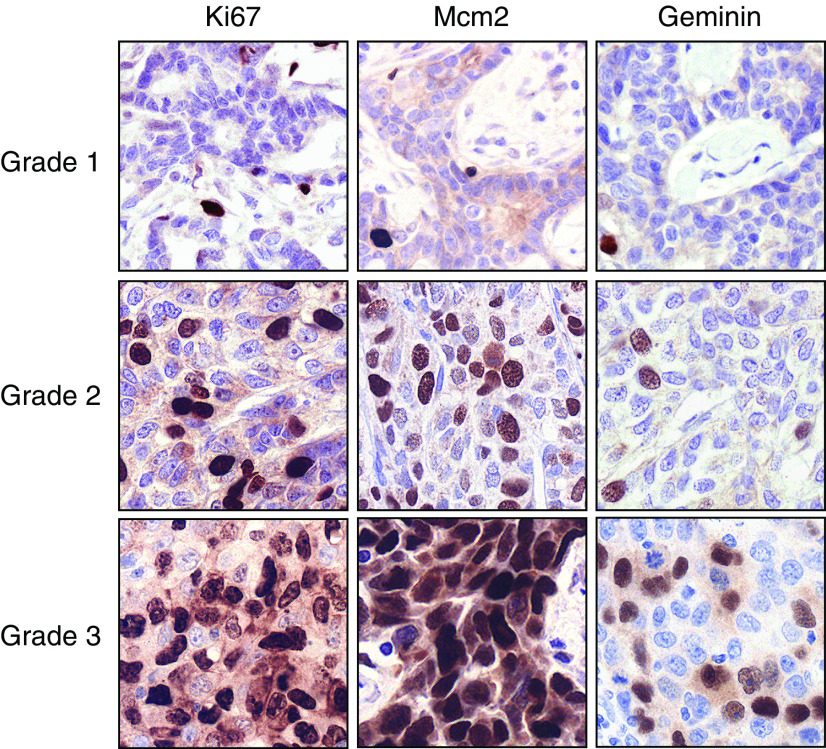
Photomicrographs of paraffin-embedded tissue sections of breast cancer grades 1–3 immunohistochemically stained with antibodies against Ki67, Mcm2 and geminin (original magnification, × 400). Increasing tumour grade is associated with increasing Ki67, Mcm2 and geminin expression. Note that Mcm2 expression is approaching 100% in grade 3 cancers.

Next we asked whether DNA replication licensing is coupled to cellular differentiation in breast cancer. To address this question, we compared Ki67, Mcm2 and geminin expression with ER and PR status in an invasive breast carcinoma series (*n*=120, Table 2). Expression profiles of replication licensing factors (RLFs) and differentiation markers were compared with tumour size, grade, lymph node status, vascular invasion and NPI. Median labelling indices for each protein are shown in Table 1 and Figure 2, and representative immunostained tissue sections are illustrated in Figure 3. As part of the analysis, Mcm2/Ki67 and geminin/Ki67 ratios were also calculated for each tumour. Both Ki67 and Mcm2 are expressed throughout all four phases of the mitotic cell cycle (G1, S, G2 and M). Mcm2–7 protein expression also identifies noncycling cells with proliferative potential (Stoeber *et al*, 2001; Eward *et al*, 2004; Wharton *et al*, 2004). The Mcm2/Ki67 ratio therefore defines the proportion of cells that are licensed to proliferate. Consequently, the higher the Mcm2/Ki67 ratio, the greater the proportion of cells that reside in a licensed noncycling state (Eward *et al*, 2004; Wharton *et al*, 2004).

Increasing grade of tumour was associated with significantly increased expression of Ki67, Mcm2 and geminin, and lower expression of the differentiation markers ER and PR (Jonkheere-Terpstra test, *P*<0.001 in each case; Table 1 and Figure 2). The decrease in the Mcm2/Ki67 ratios with increasing grade reflects a shift in the tumour cell population from a predominantly nonproliferating licensed state in differentiated tumours to an actively cycling state in poorly differentiated tumours (median grade 1: 4.13, grade 2: 2.56, grade 3: 1.85; *P*<0.001). As in the case of well-differentiated tumours, normal breast has a similarly high Mcm2/Ki67 ratio (median: 11.16) indicative of a licensed nonproliferating state. The shift from the latter state to the proliferating state with increasing tumour grade is also reflected by the increase in geminin expression, indicating that cells are engaging in DNA synthesis and progressing through S–G2–M phase (median grade 1: 2.00%, grade 2: 6.48%, grade 3: 17.14%; *P*<0.001). Higher tumour grade is also associated with an increase in the geminin/Ki67 ratio, signifying a shortening of G1 phase for the more malignant phenotype (grade 1: 0.29 *vs* grade 3: 0.36; *P*=0.07) and an increase in HER-2 expression (*P*=0.05). Interestingly, higher expression levels of geminin were observed in invasive ductal carcinoma compared to lobular carcinoma (median: 12.41 *vs* 7.14%; *P*<0.02).

A logistic regression model shows that none of the markers including Ki67, Mcm2, geminin, ER, PR, HER-2, nor the Mcm2/Ki67 or geminin/Ki67 ratios, have any statistically significant association with lymph node metastasis (Table 3). Moreover, the low values of Spearman's correlation coefficient indicate that there is no association between tumour size and any of these markers (Table 4). The results of the Mann–Whitney test indicate that presence of LVI is associated with decreased expression of ER (*P*=0.02) and PR (*P*=0.05) but no association with the other markers.

Linear regression models indicate that Ki67, Mcm2 and the Mcm2/Ki67 ratio are statistically significantly associated with the surrogate outcome measure NPI, but that this association does not apply to geminin or the geminin/Ki67 ratio. The Ki67 and Mcm2 labelling indices and the Mcm2/Ki67 ratio are highly correlated with one another, with Mcm2 being the single most important predictor of NPI score (*P*<0.001). Importantly, only 12% in the variation of NPI is explained by this marker (the value of *R*^2^ from the linear regression model equal to the square of Pearson's correlation coefficient between NPI and Mcm2), a consequence of the very high levels of Mcm2 expression in higher-grade tumours (median grade 3: 96.91%; Table 1; Figure 2).

## DISCUSSION

We and others have demonstrated that repression of origin licensing is a powerful downstream mechanism by which metazoan cells lower proliferative capacity (Yoshida *et al*, 1982; Stoeber *et al*, 2001; Blow and Hodgson, 2002; Eward *et al*, 2004). Withdrawal of cells from the mitotic cell cycle into quiescent (G0), terminally differentiated or senescent ‘out-of-cycle’ states is coupled to downregulation of Mcm2–7 and the origin licensing repressor geminin (Stoeber *et al*, 2001; Eward *et al*, 2004; Wharton *et al*, 2004). Expression profiling of these RLFs in normal premenopausal breast revealed an unusual replication phenotype. Although the growth fraction identified by the standard proliferation marker Ki67 is small, a large number of mammary epithelial cells within the TDLU express Mcm2, indicating that a large number of cells appear to be licensed and therefore ‘in-cycle’. Failure of these licensed cells to progress through the cell cycle is confirmed in this study through their failure to express the S–G2–M marker geminin. We have previously reported that during pregnancy the proportion of MCM-expressing lobular cells increases to greater 95%, coinciding with cell expansion and also including the myoepithelial cell population. This was followed by dramatic downregulation of the MCM licensing factors as cells entered the terminally differentiated lactating phenotype (Stoeber *et al*, 2001). It thus appears that persistence of Mcm2–7 expression in nonproliferating breast may be an evolutionary adaptation allowing a rapid response to pregnancy, and it remains to be determined whether this primed state may predispose the breast to genotoxic insult and facilitate the transition to uncontrolled cell proliferation. In this context it is noteworthy that the prophylactic mastectomy specimen for BRCA1 showed higher levels of Mcm2 expression than the reduction mammoplasty specimens (Figure 1B, case 1).

Our analysis of the DNA replication licensing pathway in breast cancer has revealed that MCM expression is coupled to the differentiation status of tumours. The hierarchical stem cell model of tumour growth proposes that neoplasms represent stem cell systems in which a minority of cells has the proliferative capacity to maintain the tumour, whereas the majority demonstrate features of differentiation and have limited proliferative potential (Reya *et al*, 2001; Clarke *et al*, 2003; Dontu *et al*, 2003). We have demonstrated that loss of proliferative capacity during early stages of differentiation is linked to downregulation of Cdc6, Cdt1, geminin and Ki67. In contrast, downregulation of Mcm2–7 is a late event when cells enter the terminally differentiated state (Eward *et al*, 2004). Thus, combinatorial analysis of RLFs and Ki67 allows identification of three distinct replication phenotypes: proliferating cells (Cdc6, Cdt1, Mcm2–7, geminin and Ki67 positive), differentiating cells (Mcm2–7 positive; Cdc6, Cdt1, geminin and Ki67 negative) and terminally differentiated cells (Cdc6, Cdt1, Mcm2–7, geminin and Ki67 negative). The particular replication phenotype observed in breast cancer is entirely in keeping with the hierarchical stem cell model of tumour growth, the uncoupling of Mcm2 expression with geminin and Ki67 expression increasing with a higher degree of differentiation.

We have recently shown that geminin expression provides an estimate of the S–G2–M growth fraction in dynamic cell populations, and that the geminin/Ki67 ratio defines the relative length of G1 (Eward *et al*, 2004). Proliferating cells with a short G1 phase will approximate to a geminin/Ki67 ratio of ∼1, whereas cells with a prolonged G1 will approximate to a ratio of ∼0 (Wharton *et al*, 2004). Interestingly, we observed a nonsignificant trend in the geminin/Ki67 ratio between grade 1, grade 2 and grade 3 tumours, with increasing values signifying an accelerated G1 phase in the more poorly differentiated tumours (Table 1). The lower levels of geminin expression in lobular *vs* ductal carcinoma suggest a higher rate of cell cycle progression in tumours of the latter type.

We have previously reported that the replication licensing pathway is deregulated early in epithelial carcinogenesis, and that the increased growth fraction detected by Mcm2–7 antibodies compared to Ki67 antibodies can be exploited for cancer detection and surveillance (Williams *et al*, 1998; Freeman *et al*, 1999; Stoeber *et al*, 1999, 2002; Wharton *et al*, 2001; Going *et al*, 2002). We and others have also shown that MCM proteins can provide prognostic information in a range of epithelial neoplasms including prostate, kidney and bladder (Meng *et al*, 2001; Rodins *et al*, 2002; Kruger *et al*, 2003; Dudderidge *et al*, 2005). Recently, (Gonzalez *et al* (2003) have reported that Mcm2 is a potentially useful and strong independent prognostic factor in breast cancer. In keeping with our study, the authors showed that the Mcm2 LI has a positive association with histological grade and NPI, but not lymph node stage or LVI. They also noted that the Mcm2 LI is a superior prognostic marker compared to the Ki67 LI. These findings are confirmed in our study in which we identified Mcm2 as the single most important predictor of the surrogate outcome measure NPI. However, we noted that only 12% in the variation of the NPI is explained by this marker, a consequence of Mcm2 expression reaching saturation levels in high-grade tumours. The small value of 12% suggests that, although there is a statistically significant positive association between Mcm2 and NPI, there is considerable variability in NPI that cannot be ascribed to expression of Mcm2 and so the data argue that the usefulness of Mcm2 as a surrogate measure for NPI is limited. This major constraint in the clinical utility of Mcm2 as a prognostic marker in breast cancer is also reflected in the fact that Gonzalez *et al* found the Mcm2 LI not superior to the NPI, the present gold standard prognostic indicator. We have previously demonstrated that the Mcm2–7 subunits of the hexameric MCM complex show identical cell cycle regulation and are downregulated with similar kinetics during withdrawal from the proliferative cell cycle (Stoeber *et al*, 2001). Thus, our data suggest that Mcm2–7 proteins are severely constrained as prognostic markers in breast cancer. Interestingly, we have noted in a separate study that downregulation of Mcm2 and geminin is linked to hormonal blockade and clinical response, suggesting that although these biomarkers appear to be of limited value for prognostication, they may have potential as predictors of therapeutic response in breast cancer (Shetty *et al*, unpublished data).

In summary, our investigation of the DNA replication licensing pathway has provided new insights into the cell cycle kinetics of normal and neoplastic breast, findings also of importance for potential therapeutic interventions targeting the DNA replication licensing machinery (Shreeram *et al*, 2002; Shreeram and Blow, 2003; Yoshida *et al*, 2004). Our study has shown that although MCM proteins provide prognostic information in breast cancer, these markers are likely to be weak predictors of outcome due to their high-level expression in poorly differentiated tumours.

## Figures and Tables

**Table 1:** Marker expression (median percentage of cells positive) across different tumour grades

**Marker**	**Normal tissue**	**Grade 1**	**Grade 2**	**Grade 3**
Ki67	2.14	10.26	17.11	43.66
Mcm2	35.32	55.17	70.16	96.91
Geminin	0.76	2.00	6.48	17.14
ER	22.13	100	100	13.88
PR	19.60	100	98.11	0
Mcm2/Ki67	11.16^a^	4.13	2.56	1.85
Gem/Ki67	0.46^a^	0.29	0.32	0.36

a Figures unreliable as Ki67 staining % is low.

**Table 2:** Clinicopathological details of invasive breast cancer series

**Clinicopathological feature**	**Frequency**
*Age*	
<40	12
40–59	54
>60	54

*Size (mm)*	
<11	12
11–20	36
21–30	35
31–40	17
>40	16
Unknown	4

*Lymph node stage*	
No	63
Yes	49
Unknown	8

*Grade*	
1	12
2	53
3	55

*NPI score*	
<3.4	22
3.4–5.4	62
>5.4	28
Unknown	8

*Lymphovascular invasion*	
Absent	69
Present	45
Unknown	6

*Tumour type*	
Invasive ductal	93
Lobular	20
Mucinous	4
Mixed	3

**Table 3:** Relationship between median marker expression and lymph node metastasis

	**Lymph node metastasis**	
**Marker**	**No**	**Yes**
Ki67	32.42	36.35
Mcm2	68.74	75.01
Geminin	11.52	13.10
ER	74.75	62.59
PR	60.40	50.45
Mcm2/Ki67	4.15	3.23
Gem/Ki67	0.43	0.41

**Table 4:** Correlation between markers and tumour size using Spearman's correlation coefficient

**Marker**	**Correlation**
Ki67	0.10
Mcm2	0.11
Geminin	0.09
ER	−0.03
PR	−0.02
Mcm2/Ki67	−0.01
Gem/Ki67	−0.01
